# Supplementary material for: Basic neonatal resuscitation skills of midwives and nurses in Eastern Ethiopia are not well retained: An observational study
Source: PLoS One. 2020 Jul 24;15(7):e0236194. doi: 10.1371/journal.pone.0236194 (PMC7380629; doi:10.1371/journal.pone.0236194)
Supplement: S1 File — (DOCX) [file pone.0236194.s002.docx]

**English version of the data collection tool (questionnaire and checklist)**

Basic neonatal resuscitation skills of midwives and nurses in Eastern Ethiopia are not well retained: an observational study.

Date: __________________

Interviewer name: _________________

| **No.** | **Question** | **Response** | **Code** | |
| --- | --- | --- | --- | --- |
|  | **Part I: Characteristics of health institution** | |  | |
| 101 | Code of the questionnaire? | _______ |  | |
| 102 | Health Institution’s code? | _______ |  | |
| 103 | Administrative office name? | Dire Dawa health office | 1 | |
|  |  | Harari health office | 2 | |
|  |  | East Hararge health office | 3 | |
|  |  | West Hararge health office | 4 | |
| 104 | Types of the health institution? | Referral/Specialized hospital | 1 | |
|  |  | General Hospital | 2 | |
|  |  | Primary Hospital | 3 | |
|  |  | Health Center | 4 | |
| 105 | Case load in the institution in number |  |  | |
|  | No. of deliveries in past 3 months? | _________ |  | |
|  | No. of newborn resuscitated in past 3 months? | _________ |  | |
| 106 | Is there NR corner in the institution? (*observe availability*) | Yes | 1 | |
|  |  | No | 2 | |
| 107 | Guidelines/algorisms available in the institution for newborn care? *(Observe availability and encircle those available)* | Management of newborn complication | 1 | 0 |
|  |  | Integrated Management of Newborn and Childhood Illness (IMNCI) | 1 | 0 |
|  |  | Postpartum/postnatal care of newborns | 1 | 0 |
|  |  | Immediate newborn care | 1 | 0 |
| 108 | Which of the following essential items is/are available in the institution? *(Observe availability and encircle those available)* | Functional mucus extractor | 1 | 0 |
|  |  | Functional infant ambu bag | 1 | 0 |
|  |  | Functional infant face masks (sizes 0,1,2) | 1 | 0 |
|  |  | Towels or cloth for newborn | 1 | 0 |
|  |  | Newborn resuscitation table | 1 | 0 |
|  |  | Functional clock | 1 | 0 |
|  |  | Cape | 1 | 0 |
|  |  | Pillow | 1 | 0 |
|  |  | Functional stethoscope for newborns | 1 | 0 |

| **No.** | **Question** | **Response** | **Code** | **Remark** |
| --- | --- | --- | --- | --- |
| **Part II: Socio demographic and other provider characteristics assessment question** | | | | |
| **Socio demographic characteristics assessment questions** | | | | |
| 201 | What is your age in full years? | _______ (in complete year) |  |  |
| 202 | What is the sex of the participant? | Female | 1 |  |
|  |  | Male | 2 |  |
| 203 | Marital status of the participant? | Never married | 1 |  |
|  |  | Married | 2 |  |
|  |  | Divorced/Separated | 3 |  |
|  |  | Cohabiting | 4 |  |
|  |  | Widowed | 5 |  |
| 204 | Religion of the participant? | Orthodox | 1 |  |
|  |  | Muslim | 2 |  |
|  |  | Protestant | 3 |  |
|  |  | Wakefeta | 4 |  |
|  |  | Catholic | 5 |  |
|  |  | Other (Specify) ___________ | 6 |  |
| 205 | What is your profession? | Midwifery | 1 |  |
|  |  | Nurse | 2 |  |
| 206 | What is your level of education | Diploma | 1 |  |
|  |  | BSc Degree | 2 |  |
|  |  | MSc and above | 3 |  |
| 207 | What is your experience in complete years? | _________ |  |  |
| 208 | Current practicing unit | Obstetrics & gynecology | 1 |  |
|  |  | Pediatrics & Neonatology | 2 |  |
| **Other provider characteristics assessment questions of study participants**. | | | | |
| 209 | Have you received newborn resuscitation training? | Yes | 1 |  |
|  |  | No | 2 |  |
| 210 | Have you ever performed NR? | Yes | 1 |  |
|  |  | No | 2 |  |
| 211 | Have you ever worked in delivery room | Yes | 1 |  |
|  |  | No | 2 | If 2 skip to 214 |
| 212 | Preparation for resuscitation before birth (frequency)? | At every birth | 1 |  |
|  |  | Occasionally | 2 |  |
|  |  | Never | 3 |  |
| 213 | Frequency of practicing in self-evaluation after delivery on NR? | At every birth | 1 |  |
|  |  | Occasionally | 2 |  |
|  |  | Never | 3 |  |
| 214 | Have you ever worked in neonatology/pediatrics room | Yes | 1 |  |
|  |  | No | 2 |  |
| 215 | Do you attend review meeting/morning session? | Yes | 1 |  |
|  |  | No | 2 |  |
| 216 | Frequency of practicing peer evaluation on NR? | For every resuscitation | 1 |  |
|  |  | Occasionally | 2 |  |
|  |  | Never | 3 |  |

| **No.** | **Questions** | **Correct responses** | **Code** | | **Remark** |
| --- | --- | --- | --- | --- | --- |
| **Part III: Knowledge assessment questions of study participants.**  **N.B. *For data collectors, don’t read the choice, ask open ended question and encircle the code number if participant stated correct answer 1; if not 0.*** | | | | | |
| **301** | **How would you diagnose birth asphyxia?** | Depressed breathing | **1** | **0** |  |
|  |  | Heart rate below 100 beats per minutes | **1** | **0** |  |
|  |  | Floppiness | **1** | **0** |  |
|  |  | Central cyanosis (blue tongue) | **1** | **0** |  |
| **302** | **What are the preliminary steps of newborn resuscitation? Tell me in a sequential order.** | Call for help | **1** | **0** |  |
|  |  | Explain to mother condition of baby | **1** | **0** |  |
|  |  | Place newborn face up | **1** | **0** |  |
|  |  | Wrap or cover baby, except face and upper portion of chest | **1** | **0** |  |
|  |  | Position head so neck is slightly extended | **1** | **0** |  |
|  |  | Aspirate mouth then nose | **1** | **0** |  |
|  |  | Check the abubag to be functional | **1** | **0** |  |
|  |  | Start ventilation using bag and mask | **1** | **0** |  |
| **303** | **Were the steps mentioned in sequential order? *(by data collector’s observation only)*** | Yes/No | **1** | **0** |  |
| **304** | **What do you do when resuscitating with a bag and mask or tube and mask?** | Place mask to cover chin, mouth and nose | **1** | **0** |  |
|  |  | Ensure seal between mask and face | **1** | **0** |  |
|  |  | Ventilate 1 or 2 times and see if chest is rising | **1** | **0** |  |
|  |  | Ventilate 40 times per minute for 1 minute | **1** | **0** |  |
|  |  | Pause to determine whether baby is breathing spontaneously | **1** | **0** |  |
| **305** | **What do you do if the baby is breathing and there is no sign of respiratory difficulty?** | Keep baby warm | **1** | **0** |  |
|  |  | Initiate breastfeeding | **1** | **0** |  |
|  |  | Continue monitoring the baby | **1** | **0** |  |
| **306** | **What do you do if the baby does not begin breathing, breathing is < 30/minute, or if there is intercostals retraction or grunting?** | Continue to ventilate for 20 minutes | **1** | **0** |  |
|  |  | Administer oxygen, if available | **1** | **0** |  |
|  |  | Assess the need for special care | **1** | **0** |  |
|  |  | Explain to mother what is happening | **1** | **0** |  |
|  |  | Refer the baby to higher level care service | **1** | **0** |  |

| **No.** | **Tasks** | **Observation** | | | **Remark** |
| --- | --- | --- | --- | --- | --- |
|  |  | **2** | **1** | **0** |  |
| **Part IV: Observational checklist for study participant during simulation of newborn resuscitation.**  **N.B. Encircle “2” if step/task is performed satisfactorily, “1” if it is not performed satisfactorily, or “0”if not observed. *(Some of the steps/tasks should be performed simultaneously.)*** | | | | | |
| 401 | **Getting ready** |  |  |  |  |
|  | Make sure equipment is ready for use | 2 | 1 | 0 |  |
|  | Wash hands and wear gloves | 2 | 1 | 0 |  |
|  | Quickly dry and wrap or cover the newborn | 2 | 1 | 0 |  |
|  | Place newborn on back on clean, warm surface | 2 | 1 | 0 |  |
|  | Tell woman what is going to be done, listen to her, and respond to her questions and concerns | 2 | 1 | 0 |  |
|  | Provide emotional support and reassurance | 2 | 1 | 0 |  |
| 402 | **Resuscitation using bag and mask** |  |  |  |  |
|  | Position head in slightly extended position | 2 | 1 | 0 |  |
|  | First introduce catheter into mouth and suction | 2 | 1 | 0 |  |
|  | Second introduce catheter into each nostril and suction | 2 | 1 | 0 |  |
|  | If baby is still not breathing, start ventilating | 2 | 1 | 0 |  |
|  | Recheck position of newborn’s head | 2 | 1 | 0 |  |
|  | Recheck ambubag | 2 | 1 | 0 |  |
|  | Place correct-sized mask on newborn’s face | 2 | 1 | 0 |  |
|  | Form a seal between mask and newborn’s face | 2 | 1 | 0 |  |
|  | Squeeze bag | 2 | 1 | 0 |  |
|  | Check seal by ventilating and observing chest rise | 2 | 1 | 0 |  |
|  | **If the newborn’s chest IS rising:** |  |  |  |  |
|  | Ventilate at 40 breaths/minute every 1 minute for 20 minute | 2 | 1 | 0 |  |
|  | Observe chest for easy rise and fall 2 to 3 times. | 2 | 1 | 0 |  |
|  | **If the newborn’s chest IS NOT rising:** |  |  |  |  |
|  | Check position of the head again | 2 | 1 | 0 |  |
|  | Reposition mask to improve seal | 2 | 1 | 0 |  |
|  | Repeat suction | 2 | 1 | 0 |  |
|  | Ventilate for 1 minute and then assess if the newborn is breathing spontaneously **(>30b/min**) | 2 | 1 | 0 |  |
|  | **If breathing is normal (no indrawing or grunting):** |  |  |  |  |
|  | Place in skin-to-skin contact with mother | 2 | 1 | 0 |  |
|  | Observe breathing at frequent intervals | 2 | 1 | 0 |  |
|  | Encourage mother to begin breastfeeding | 2 | 1 | 0 |  |
|  | **If newborn is breathing with severe indrawing (<30b/min):** |  |  |  |  |
|  | Ventilate with oxygen, if available | 2 | 1 | 0 |  |
|  | Arrange immediate transfer for special care | 2 | 1 | 0 |  |
|  | If there is no gasping or breathing at all after 20 minutes of ventilation, stop ventilating | 2 | 1 | 0 |  |
| 403 | **Post**-**procedure tasks** |  |  |  |  |
|  | Place disposable suction catheters and mucus extractors in leak-proof container | 2 | 1 | 0 |  |
|  | **For reusable catheters and mucus extractors:** |  |  |  |  |
|  | Place in chlorine solution for 10 minutes | 2 | 1 | 0 |  |
|  | Wash in water and detergent | 2 | 1 | 0 |  |
|  | Use a syringe to flush catheters/tubing | 2 | 1 | 0 |  |
|  | Boil or disinfect in chemical solution | 2 | 1 | 0 |  |
|  | Take apart valve/mask and inspect for cracks/tears | 2 | 1 | 0 |  |
|  | Wash valve/mask and check for damage | 2 | 1 | 0 |  |
|  | Select sterilization or high-level disinfection method | 2 | 1 | 0 |  |
|  | Wash hands and dry with clean cloth or air dry | 2 | 1 | 0 |  |
|  | After chemical disinfection, rinse all parts with clean water and allow to air dry | 2 | 1 | 0 |  |
